# MSC Origin and Biomechanical Conditioning Determine ECM Maturation in Tissue-Engineered Matrix

**DOI:** 10.3390/biomedicines14030560

**Published:** 2026-02-28

**Authors:** Michelle Klein, Arian Ehterami, Neguin Ranjbar, Simon P. Hoerstrup, Maximilian Y. Emmert, Melanie Generali

**Affiliations:** 1 Institute for Regenerative Medicine (IREM), University of Zurich, Wagistrasse 12, 8952 Schlieren, Switzerland; 2 Department of Cardiothoracic and Vascular Surgery, Deutsches Herzzentrum der Charité (DHZC), 13353 Berlin, Germany; 3 Charité—Universitätsmedizin Berlin, Corporate Member of Freie Universität Berlin and Humboldt-Universität zu Berlin and Berlin Institute of Health, 13353 Berlin, Germany; 4 BIH Center for Regenerative Therapies (BCRT), Berlin Institute of Health at Charité-Universitätsmedizin Berlin, 13353 Berlin, Germany; 5 Department of Biosciences, University of Milan, 20133 Milan, Italy; 6 German Center for Cardiovascular Research (DZHK), 13353 Berlin, Germany

**Keywords:** mesenchymal stromal cells, extracellular matrix, tissue engineering, collagen maturation, hydrodynamic stimulation

## Abstract

**Background**: The extracellular matrix (ECM) plays a central role in the mechanical strength and functional integration of tissue-engineered matrix (TEM), particularly in cardiovascular and load-bearing applications. Mesenchymal stromal cells (MSCs) from different sources may vary in their ECM-forming potential. **Methods**: In this study, adipose-derived (hADMSC), bone marrow-derived (hBMSC), and umbilical cord-derived MSCs (hUCMSC) were compared with human dermal fibroblasts (HDFBs) as a reference. Cells were seeded onto polyglycolic acid (PGA)/poly-4-hydroxybutyrate (P4HB) scaffolds and cultured for 3 weeks under static or hydrodynamic conditions using orbital shaking. TEM development was assessed macroscopically, histologically (using H&E and Masson’s trichrome stains), and by polarized light microscopy (Picrosirius Red), alongside biochemical assays that quantified DNA, glycosaminoglycan (GAGs), and hydroxyproline (HYP). **Results**: Hydrodynamically stimulated culture consistently improved ECM deposition across all groups. TEMs exposed to hydrodynamic stimulation (hydrodynamic conditions) were thicker, more uniformly filled, and exhibited increased collagen deposition compared with static TEMs, which remained thinner and showed persistent scaffold remnants. Polarized light analysis demonstrated that dynamic loading promoted collagen maturation in all groups, as evidenced by an increased prevalence of thick, birefringent collagen fibers indicative of mature collagen. Biochemical analyses showed that HDFB-derived TEMs produced the highest total collagen and ECM content under both static and hydrodynamic conditions; however, these matrices remained comparatively thin and densely packed. In contrast, MSC-derived TEMs formed thicker and more spatially distributed ECM in response to hydrodynamic stimulation. **Conclusion**: Among the MSC sources, hUCDMSC-derived TEMs exhibited the most advanced collagen maturation and the most uniform collagen distribution under hydrodynamically stimulated culture, whereas hADMSC-derived TEMs showed the greatest matrix thickening and volumetric ECM expansion with intermediate collagen maturation. hBMSC-derived TEMs displayed clear responsiveness to hydrodynamic stimulation but remained limited in overall collagen deposition and fiber maturation. These findings underscore that both hydrodynamic stimulation and cell source are critical not only for maximizing ECM deposition, but also for ensuring physiologically relevant collagen maturation and matrix organization in grafts suitable for clinical translation.

## 1. Introduction 

The extracellular matrix (ECM) is a complex three-dimensional network of macromolecules, including collagens, elastin, proteoglycans, hyaluronan, and diverse glycoproteins, that surrounds cells in all tissues [1]. Far from being an inert scaffold, the ECM provides not only structural support but also a dynamic biochemical environment that regulates cell behavior, influencing processes such as adhesion, migration, proliferation, and differentiation [2]. Cells interact with the ECM via receptors such as integrins, which physically link the ECM to the cytoskeleton and transmit mechanical and chemical signals that direct cell fate and tissue homeostasis [3]. The composition and architecture of the ECM are finely tuned to each tissue’s functional demands; for example, fibrillar collagens impart tensile strength, elastin confers elasticity, and hydrated proteoglycans resist compressive forces, together defining the mechanical properties of the tissue [4,5]. Importantly, not only the amount of ECM but also its molecular organization and collagen maturation, including fibril alignment, crosslinking, and transition from immature to mature collagen fibers, determine the mechanical integrity and functionality of the matrix [6,7].

In state-of-the-art tissue engineering (TE) and regenerative medicine, ECM-based scaffolds have become foundational for creating functional tissues [8]. Decellularized ECM (dECM) materials, produced by removing cells from donor tissues, retain native biochemical cues and 3D ultrastructure, providing a bioactive microenvironment that promotes cell attachment, proliferation, and differentiation [9]. Clinically, multiple ECM-derived grafts are already in use; decellularized human dermal matrices (AlloDerm^®^, GraftJacket^®^) are applied as skin grafts, and porcine small-intestinal submucosa (OASIS^®^) is used as a bioactive wound dressing substitute [10,11,12]. Beyond clinical uses, ECM plays a significant role in biomedical research and pharmaceutical development [5,13,14]. Inspired by the intricate structure and composition of native ECM, next-generation biomaterials are being engineered to replicate not only its biochemical signals but also its multi-scale architecture, enabling enhanced cell signaling, immune modulation, and tissue remodeling in regenerative applications [15]. These studies highlight how ECM-based platforms bridge in vitro research and clinical therapy by providing realistic 3D tissue models for drug screening and disease modelling, and by enabling localized delivery of therapeutics.

Different cell types vary greatly in their capacity and manner of ECM production, reflecting the specialized needs of their resident tissues [16,17,18]. Connective tissue fibroblasts are prolific producers of interstitial ECM, secreting abundant type I collagen, fibronectin, and other matrix components to form the structural framework of skin, tendons, and other fibrous tissues [19]. Chondrocytes, by contrast, are sparsely distributed cells that maintain cartilage; they synthesize a rich matrix dominated by type II collagen and large aggregating proteoglycans (e.g., aggrecan), which together provide cartilage with its load-bearing, compressive resilience [20,21]. Osteoblasts in bone deposit a dense collagenous matrix (primarily type I collagen) that becomes mineralized with hydroxyapatite, creating the rigid, weight-bearing scaffold of the skeleton [22]. In general, tissues like articular cartilage and fibrocartilage are highly ECM-rich (with few cells and extensive matrix), whereas others (e.g., epithelial tissues) have minimal ECM confined to basement membranes. Human mesenchymal stromal cells (MSCs) are multipotent stromal cells capable of differentiating into osteogenic, chondrogenic, adipogenic, and other mesenchymal lineages [2,23]. MSCs play a key role in tissue repair and regeneration, not only by differentiating into tissue-forming cells but also by actively secreting ECM components and growth factors that orchestrate the healing microenvironment [24]. This makes MSCs an attractive cell source for TE strategies aimed at regenerating or replacing damaged tissues with the help of a robust ECM produced by the cells.

The environmental conditions and mechanical cues to which cells are exposed have a profound influence on their ECM production. In static in vitro cultures, cells often deposit ECM at a limited rate and with a disorganized architecture, partly due to the absence of biomechanical signals present in vivo [25,26]. In contrast, flow-induced culture conditions, such as orbital shaking that generate hydrodynamic stimulation and enhance nutrient and waste transport, markedly enhance ECM deposition while also promoting collagen maturation and fibril organization [27].

A high level of ECM deposition is particularly critical for load-bearing tissues, as the ECM is the primary determinant of mechanical strength and functionality. Equally important, the degree of collagen maturation, including fibril alignment, crosslinking, and hierarchical organization, plays a decisive role in defining ECM quality, mechanical performance, and the durability of TEM. In this study, hydrodynamic stimulation was achieved using orbital shaking, which does not reproduce defined tensile or compressive loading modes but provides continuous fluid-induced shear stress, improved mass transport, and low-level mechanical stimulation that can activate mechanosensitive pathways involved in ECM synthesis. Biodegradable scaffolds are referred to as “scaffolds” before cell seeding and culture, and as “TE matrix (TEM)” following in vitro conditioning to reflect the presence of newly deposited ECM. Accordingly, we aim to evaluate ECM quantity and quality produced by human umbilical cord MSCs (hUCMSCs), human bone marrow MSCs (hBMSCs), and human adipose-derived MSCs (hADMSCs) in a TE context, while comparing static and hydrodynamic stimulated culture conditions for MSC-seeded scaffolds and human dermal fibroblasts (HDFBs).

## 2. Materials & Methods

### 2.1. Cell Culture

#### 2.1.1. Culture of Human Dermal Fibroblasts

HDFBs were obtained from CellSystems Biotechnology GmbH (Troisdorf, Germany) as commercially available cells. These cells were used as a reference cell population for comparative analyses. Cells were maintained at 37 °C in a humidified atmosphere with 5% CO_2_ using advanced DMEM (Sigma, Buchs, Switzerland) supplemented with 10% fetal bovine serum, 1% GlutaMAX (Gibco, Grand Island, NY, USA), and 1% penicillin/streptomycin (Sigma, Switzerland). The culture medium was replaced every third day, and cells were passaged using trypsin (Innovative Cell Technologies Inc., San Diego, CA, USA) upon reaching confluence.

#### 2.1.2. Culture of Human MSCs

hMSCs were obtained from three tissue sources: hADMSCs, hBMSCs, and hUCMSCs. Each cell line was isolated from a unique donor following previously described methods (Supplementary Table S1) [28]. The Cantonal Ethics Committee Zurich granted ethical approval for human tissue procurement and MSC isolation (hADMSCs: KEK-ZH-2010-0476; hUCMSCs: KEKZH-2009-0095). hBMSCs were obtained under institutional approval according to standard procedures. Cells were maintained at 37 °C in a humidified atmosphere with 5% CO_2_ using advanced DMEM (Sigma, Switzerland) supplemented with 10% fetal bovine serum, 1% GlutaMAX (Gibco, USA), and 1% penicillin/streptomycin (Sigma, Switzerland). The culture medium was replaced every third day, and cells were passaged using trypsin (Innovative Cell Technologies Inc., USA) upon reaching confluence.

### 2.2. Proliferation Assay

Cell proliferation was quantified by crystal violet staining. Cells were seeded at 500 per well in 96-well plates and cultured for up to seven days. Each day, cells were fixed with methanol (Sigma-Aldrich, Buchs, Switzerland), stained with 0.1% crystal violet (Artechemis, Zofingen, Switzerland), washed, and air-dried. Bound dye was solubilized in 2% sodium deoxycholate (Sigma-Aldrich, Buchs, Switzerland) at 60 °C, and absorbance was measured at 550 nm using a Synergy HT reader (BioTEK, Winooski, VT, USA). Quantification was performed against a standard curve of serial dilutions.

### 2.3. ECM Production

Biodegradable non-woven polyglycolic acid (PGA) meshes (thickness 1.0 mm; Cellon, Oslo, Norway) coated with poly-4-hydroxybutyrate (P4HB; TEPHA Inc., Lexington, MA, USA), which was dissolved in liquid tetrahydrofuran (Sigma-Aldrich, St. Louis, MO, USA), were used as starter scaffolds for the generation of TEMs, following previously established protocols [29,30]. Briefly, coated scaffolds were mounted onto plastic rings (28 mm diameter) prior to cell seeding. HDFBs (*n* = 6) and three distinct human MSC populations (hUCMSCs, hBMSCs, hADMSCs; *n* = 6 per group) were seeded at a density of 1.5 × 10^6^ cells/cm^2^. Seeded scaffolds were incubated for 2 days under static conditions in Advanced DMEM supplemented with 10% fetal bovine serum, 1% penicillin/streptomycin, 1% GlutaMAX, and 0.9 mM L-ascorbic acid-2-phosphate (Sigma-Aldrich, Buchs, Switzerland). Thereafter, scaffolds were maintained either under static culture or under hydrodynamic stimulation by placement on an orbital shaker, generating fluid-induced shear stress through fluid motion and non-uniform hydrodynamic forces that result in scaffold motion.

### 2.4. Qualitative Tissue Analysis

#### 2.4.1. Macroscopic Assessment

After 21 days of culture, all TEMs were harvested and photographed to assess morphological differences. Visual inspection was performed to capture variations between the different cell types and culture conditions.

#### 2.4.2. Histology

For histological and histochemical evaluation of ECM production (*n* = 6 per group), 5 μm thick sections were prepared from formalin-fixed, paraffin-embedded tissue blocks and mounted onto glass slides (SuperFrost Plus, Menzel Gläser, Braunschweig, Germany). Sections were subsequently deparaffinized, rehydrated, and subjected to hematoxylin and eosin (H&E), Picrosirius Red, Masson’s Trichrome, and Elastica van Gieson (EvG) staining following standard protocols. To further characterize ECM components, immunohistochemical staining was performed for fibronectin. After antigen retrieval and blocking, sections were incubated with primary antibodies against fibronectin (Millipore, AB2033, Burlington, MA, USA) followed by appropriate secondary antibodies and chromogenic detection (performed by the Histology Core Facility, University Hospital Zurich [USZ]). All stained sections were analyzed using a Mirax Midi brightfield slide scanner (Zeiss, Oberkochen, Germany) and analyzed with the MIRAX viewer software (V1.11) (Zeiss, Germany).

To evaluate structural differences between groups, the thickness of each histological TEM was quantified on H&E-stained sections. For every sample, thickness was measured at five evenly distributed locations across the tissue cross-section using the digital measurement tools integrated in the MIRAX viewer software (Zeiss, Germany). The five measurements were averaged to obtain a representative thickness value per sample. Subsequently, mean thickness values were calculated for each experimental group. All measurements were performed by a blinded observer to ensure consistency and reduce measurement bias.

To assess cell density within the TEM, nuclei were quantified on H&E-stained sections. For each TEM, five non-overlapping regions of interest (ROIs) were randomly selected across the tissue cross-section. Nuclei within each ROI were manually counted by two independent observers blinded to the experimental groups, and counts were averaged to obtain a representative value per TEM. Final data are presented as mean nuclei count per section for each group.

#### 2.4.3. Polarized Light Microscopy

Picro-Sirius Red–stained sections were examined under polarized light microscopy (Keyence, Mechelen, Belgium) to visualize collagen fiber organization and birefringence. Images were acquired to distinguish between mature and immature collagen fibers based on their birefringent properties and to detect remnants of the scaffold material within the TEMs. Under polarized light, thicker and more organized collagen fibers appear yellow–orange to red, whereas thinner and less organized fibers appear green. All samples were imaged using identical polarization and exposure settings to allow direct comparison between experimental groups.

### 2.5. Quantitative Tissue Analysis

Produced ECM (*n* = 6 per group) were minced, lyophilized, and subjected to biochemical assays to quantify total DNA, hydroxyproline (HYP), and glycosaminoglycan (GAG) content as indicators of cell number, collagen deposition, and extracellular matrix composition, respectively [31,32]. Samples were lyophilized for at least 4 h and subsequently weighed to ensure a minimum dry mass of 2–3 mg per specimen. The TEMs were then digested in a papain solution (125–140 µg/mL in digestion buffer; Sigma-Aldrich, Switzerland) at 60 °C for 16 h.

Following digestion, samples were centrifuged at 12,000 rpm to remove debris and, if necessary, diluted in digestion buffer to fall within the linear range of the assay. GAG content was quantified using the 1,9-dimethylmethylene blue (DMMB) dye-binding assay [33]. Briefly, 40 µL of supernatant was transferred to 96-well plates in triplicate, mixed with 150 µL DMMB solution, and absorbance was recorded within 10 min at 540 and 595 nm. Net absorbance (540–595 nm) was compared against a standard curve prepared from serial dilutions of chondroitin sulfate. GAG concentration was calculated from the regression equation, multiplied by the respective digestion volume, and normalized to the dry weight of each TEM.

DNA concentration within the digests was quantified using the Quant-iT™ PicoGreen^®^ dsDNA assay kit (Invitrogen, Darmstadt, Germany) according to the manufacturer’s protocol [34]. Briefly, samples were mixed with PicoGreen working solution in 96-well plates, incubated for 5 min at room temperature, and protected from light. Fluorescence was recorded using a microplate reader with an excitation wavelength of 480 nm and an emission wavelength of 520 nm. Values were calculated from a standard curve prepared with λ-DNA and expressed as DNA content per TEM.

Collagen content was determined by quantifying hydroxyproline. Samples were hydrolyzed in NaOH and autoclaved at 120 °C for 10 min. Following neutralization with citric acid and centrifugation, aliquots of the hydrolysates were reacted sequentially with chloramine-T and aldehyde/perchloric acid solutions to form a chromophore [35]. Absorbance was measured at 550 nm in a microplate reader (Tecan Infinite M1000 Pro, Tecan Group Ltd., Männedorf, Switzerland). Hydroxyproline concentrations were calculated from a standard curve prepared with serial dilutions of the hydroxyproline reference solution. Values were corrected for the initial hydrolysis volume and normalized to the dry weight of each sample.

### 2.6. Statistics

Quantitative data are presented as mean ± standard deviation. Proliferation data were analyzed by two-way ANOVA with cell type (HDFBs, hUCMSCs, hADMSCs, hBMSCs) and time as independent factors, followed by post hoc multiple-comparison testing where appropriate. A *p*-value < 0.05 was considered statistically significant. Normality of the data was assessed using the Kolmogorov–Smirnov (KS) test (*p* > 0.05). All statistical analyses were performed using GraphPad Prism 10.

## 3. Results

### 3.1. Cell Morphology and Proliferation

**Figure 1:**
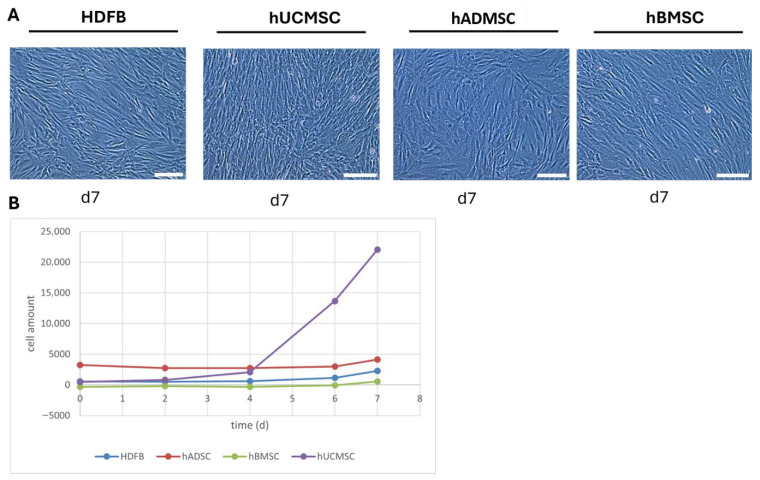
MSC morphology and proliferation. (**A**) Phase-contrast micrograph of human MSCs (representative image, 20× showing the typical spindle-shaped, fibroblast-like morphology of cultured MSCs. All three MSC sources exhibited similar cell shape and adherence. Scale bars: 50 µm. (**B**) Cell amount of MSCs and dermal fibroblasts over 7 days (mean ± SD, *n* = 3 per cell type). hUCMSC expanded more rapidly than hBMSC by day 7 (*p* < 0.05), with hADMSC and HDFB showing intermediate growth.

All three MSC populations readily attached to the cell culture plate and displayed a spindle-shaped, fibroblastic morphology characteristic of mesenchymal stem cells. In standard 2D culture, MSCs were morphologically indistinguishable from dermal fibroblasts, showing an elongated, bipolar shape with prominent cell spreading (Figure 1A). No obvious morphological differences were noted among MSC sources during expansion or immediately after seeding on scaffolds. All cell types maintained high viability and confluence before seeding, indicating robust cell quality.

MSC proliferation assays over 7 days in monolayer culture revealed source-dependent growth kinetics (Figure 1B). hUCMSCs exhibited the fastest growth, reaching a significantly higher cell number by day 7 compared to hBMSCs (*p* < 0.05). hADMSCs showed an intermediate proliferation rate, not differing significantly from hUCMSCs or hBMSCs until the final days. HDFBs proliferated at a rate comparable to hADMSCs, doubling in cell number by about day 5. By day 7, hUCMSCs had expanded more than ten-fold compared to hBMSCs, while hADMSC and HDFB yields were intermediate. Statistical analysis confirmed a significant effect of cell type on proliferation (cell type versus time interaction not significant), with hUCMSC > hADMSC ≥ HDFB > hBMSC in overall growth. These data suggest that, under identical culture conditions, hUCMSCs possess a higher proliferative capacity than adult MSCs, whereas fibroblast proliferation is within the range of MSCs.

### 3.2. Qualitative Tissue Analysis

#### Macroscopic and Microscopic Analysis

**Figure 2:**
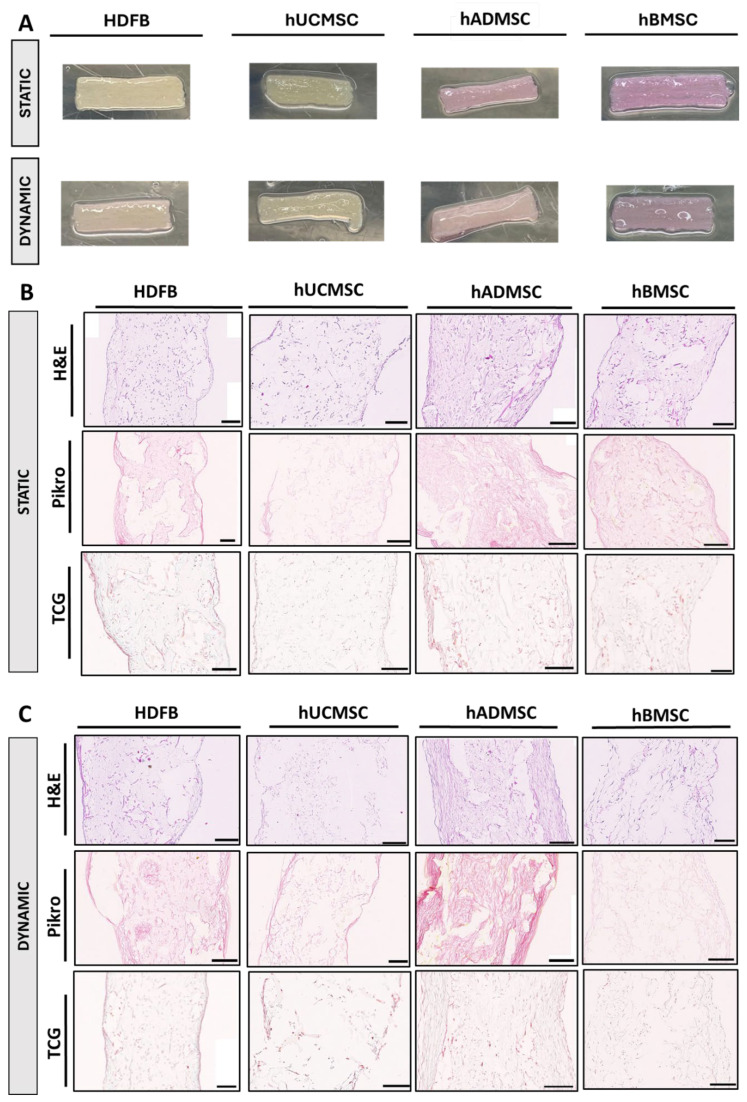
Qualitative analysis of TEMs after 21 days. Macroscopic view of representative cell-seeded TEMs under static vs. hydrodynamically stimulated culture (**A**). Hydrodynamically stimulated culture resulted in thicker, opaque tissue formation completely covering the PGA/P4HB scaffold, whereas static TEMs remained thinner and semi-translucent. TEMs were analyzed using haematoxylin and eosin (H&E), Picrosirius red (Picro) staining, and Masson’s Trichrome (TCG). Static cultures (**B**) exhibited thinner cell layers, more visible scaffold remnants, and weaker collagen staining, whereas dynamic cultures (**C**) showed denser cellular infiltration and more extensive ECM deposition. Scale bars: 100 µm.

After 21 days of culture on PGA/P4HB scaffolds, differences in ECM formation were evident between different cell types as well as between static and dynamic TEMs. Macroscopically, all TEMs formed visible tissue after 21 days (Figure 2A). Static TEMs remained relatively thin and partially translucent, with scaffold fibers still apparent. Dynamic TEMs, across all cell types, appeared thicker, opaquer, and exhibited more uniform surface coverage. This macroscopic difference was consistently observed for hADMSC-, hBMSC-, hUCMSC-, and HDFB-TEMs.

H&E confirmed clear differences in tissue distribution between static and hydrodynamically stimulated culture (Figure 2B,C). Static TEMs showed tissue primarily along the outer surface of the scaffold, with sparse matrix bridging between PGA fibers and large acellular regions remaining in the center (Figure 2B). Dynamic TEMs displayed tissue throughout the entire thickness of the TEMs, with more continuous cell- and matrix-filled areas extending from the surface toward the TEMs interior (Figure 2C).

Picrosirius Red staining demonstrated clear differences in collagen distribution between static and hydrodynamically stimulated culture (Figure 2B,C). Static MSC-derived TEMs showed sparse collagen deposition, primarily localized to the matrix surface and around individual scaffold fibers, with large unstained regions within the interior. In contrast, dynamic TEMs exhibited more continuous collagen coverage extending deeper into the matrices, with collagen filling a greater proportion of the inter-fiber spaces. Under hydrodynamic conditions, HDFB-derived TEMs displayed a dense and spatially homogeneous collagen signal, consistent with their high overall collagen content, albeit within comparatively thin matrices. In contrast, MSC-derived TEMs formed thicker matrices with a more distributed collagen network, reflecting volumetric ECM expansion rather than dense packing. Among MSCs, hUCMSC-derived TEMs showed the most extensive collagen distribution across the matrix thickness, whereas hBMSC-derived static TEMs exhibited the weakest collagen presence, limited mainly to the periphery. hADMSC-derived TEMs demonstrated an intermediate but more organized collagen network, exceeding hBMSCs while remaining less extensive than hUCMSCs. Collectively, these observations indicate that hydrodynamically stimulated culture enhances collagen deposition and penetration, while cell source governs whether collagen is organized as a dense, compact matrix (HDFBs) or as a thicker, more distributed network (MSCs), consistent with biochemical and quantitative findings.

Masson’s Trichrome staining provided complementary evidence of collagen content and distribution (Figure 2B,C). In dynamic TEMs, extensive collagen deposition was seen as broad green areas throughout the tissue, whereas static TEMs showed only fine collagen fibers, mainly at the periphery of the TEMs’ pores. Cells and cytoplasm (stained red) were intermingled with collagen, highlighting the integrated nature of the neotissue in dynamic samples. For example, HDFB dynamic TEMs were dominated by dense blue staining filling the scaffold interior, confirming a collagen-rich matrix, while HDFB static TEMs had blue staining and more red-stained cell areas, indicating less collagen overall. Similarly, hUCMSC dynamic sections showed a thick band of blue collagen matrix with embedded cells, whereas hBMSC static sections had only thin blue collagen outlines. Altogether, the histological stains qualitatively demonstrate that hydrodynamically stimulated culture markedly improves ECM formation, producing a thicker, more collagen-rich tissue with more mature collagen fibers, whereas static culture yields a scantier and less organized matrix. When comparing MSC sources, hUCMSCs again produced the most substantial collagen deposition under hydrodynamically stimulated culture, closely approaching the levels observed in HDFBs. hADMSCs formed moderately thick collagen bands but with less uniform distribution, while hBMSCs generated only sparse peripheral fibers and limited interstitial filling, even under hydrodynamic conditions. HDFBs remained the most prolific matrix producers, yielding dense and homogeneous collagen-rich tissue. These findings emphasize that cell source influences the extent of collagen deposition, with HDFBs and hUCMSCs outperforming hADMSCs and hBMSCs in neotissue quality and distribution.

In addition, elastin and fibronectin were further evaluated (Supplementary Figure S1). Elastin was assessed using EvG staining; however, elastin fibers were not detectable at this stage of TEM development. In contrast, fibronectin immunostaining revealed matrix-associated brown chromogenic signal across all groups, with a more continuous distribution observed under hydrodynamically stimulated conditions. MSC-derived matrix exhibited variable fibronectin organization, whereas HDFB-derived TEMs showed a comparatively more uniform matrix distribution.

Quantitative histological analysis demonstrated significant differences in TEM thickness across cell types and culture conditions (Table 1). Under static culture, MSC-derived TEMs were generally thicker than HDFB-derived matrices, with hADMSCs producing the greatest thickness, while hUCMSCs showed no significant difference compared to HDFBs. Notably, increased thickness did not directly correlate with collagen density, as HDFBs formed thinner yet more densely collagenized matrices, consistent with biochemical collagen quantification. Hydrodynamic stimulation further enhanced matrix thickness across MSC groups, with hADMSCs achieving the highest overall thickness and hBMSCs showing the strongest relative increase compared to static culture. In contrast, HDFBs exhibited only modest changes under hydrodynamic conditions. Overall, MSCs preferentially increased matrix thickness and volumetric ECM distribution, whereas HDFBs generated thinner but more collagen-dense matrices.

**Figure 3:**
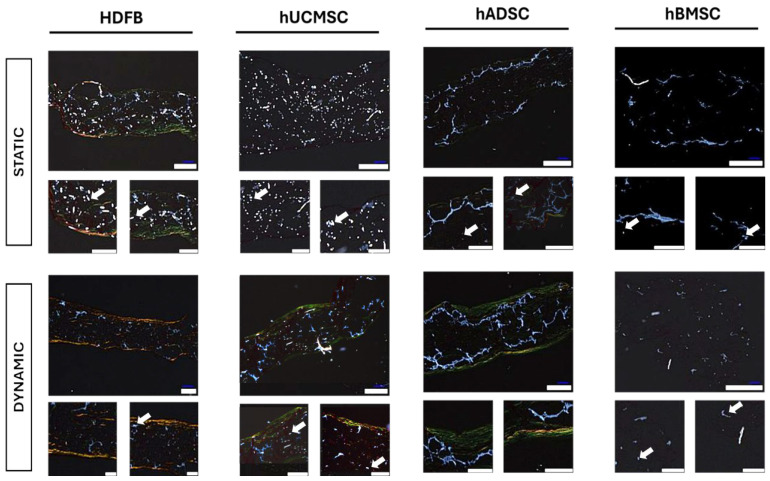
Picrosirius Red under polarized light to assess collagen fiber maturity. In the hydrodynamic condition, numerous thick collagen fibers appear orange red, indicating more mature and highly organized collagen, whereas the static condition is dominated by thin greenish fibers, indicative of immature and less organized collagen. Fewer black regions (no signal) are seen in the dynamic image, suggesting more complete tissue fill and less residual scaffold. Residual PGA fibers are shown as elongated ellipses (white arrows) (scale bars: 200 µm low magnification, 100 µm high magnification).

When TEMs were viewed under polarized light, striking differences in collagen fiber thickness, packing, and organization were observed between static and hydrodynamic conditions (Figure 3). In Picrosirius Red polarization mode, thick, highly ordered collagen fibers appear yellow-orange to red, whereas thinner, less organized, and immature collagen fibers appear green [36]. The dynamically cultured tissues displayed abundant yellow-orange birefringent fibers, indicative of more mature and thicker collagen fibers. In contrast, static cultures exhibited predominantly greenish fiber birefringence, indicating thinner and less mature collagen fibers. For instance, polarized images of hADMSC TEMs showed that dynamic samples contained a network of yellow-orange collagen fibers spanning the TEMs’ voids, whereas static samples had only sparse green collagen fibers along edges. HDFBs-seeded TEMs under dynamic loading showed large orange-red collagen bundles, reflecting a highly organized matrix. hUCMSC dynamic TEMs also demonstrated many orange fibers, whereas hBMSC static TEMs had mostly green, thin fibers. These semi-quantitative polarized light observations suggest that hydrodynamic stimulation not only increases collagen quantity but also promotes collagen fiber thickening and organization in the engineered tissue. Under polarized light, collagen-rich regions were readily identified by birefringent fibers, whereas collagen-poor areas appeared non-birefringent. In addition, residual PGA/P4HB fibers could be distinguished as optically distinct, elongated structures lacking collagen-associated birefringence. Static samples contained more collagen-poor regions and more frequent scaffold-like features, while dynamically cultured TEMs exhibited a more continuous collagen network with fewer scaffold-dominated areas, consistent with enhanced matrix deposition throughout the matrix.

### 3.3. Quantitative Tissue Analysis

**Figure 4:**
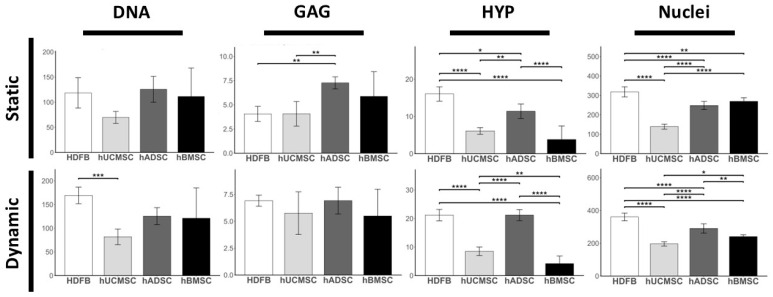
Quantitative analysis of engineered tissues after 21 days of static and dynamic culture. Biochemical assays measured: DNA content (µg/mg dry tissue) as an indicator of cell number, glycosaminoglycan (GAG) content (µg/mg dry tissue), and hydroxyproline content (HYP) (µg/mg dry tissue) as a measure of collagen deposition. Histological quantification from H&E-stained sections is shown. Data are presented as mean ± SD (*n* = 6 per group). (* *p* < 0.05, ** *p* < 0.01, *** *p* < 0.001, **** *p* < 0.0001).

Biochemical and histological quantification after 21 days demonstrated significant effects of both cell source and hydrodynamic stimulation on engineered tissue composition (Figure 4). DNA, GAG, and HYP content were quantified to assess cellularity, proteoglycan deposition, and collagen accumulation, respectively, complemented by histological nuclei counts. Across parameters, hydrodynamic stimulation generally enhanced matrix production, with cell-type-specific responses.

Under static conditions, DNA content ranged from approximately 8–14 µg/mg dry tissue across groups, with hADMSCs exhibiting the highest values (Figure 4). Hydrodynamic stimulation did not uniformly increase DNA levels; HDFBs showed the highest DNA content under dynamic culture (~16 µg/mg), whereas MSC groups remained within a comparable range (approximately 9–13 µg/mg).

GAG levels under static culture ranged between ~4–7 µg/mg dry tissue, with hADMSCs producing the highest amounts (Figure 4). Hydrodynamic stimulation modestly increased GAG deposition in most MSC groups, reaching approximately 6–7 µg/mg under dynamic conditions. HDFBs exhibited comparatively stable GAG levels between static and hydrodynamic culture.

Collagen deposition showed the most pronounced response to hydrodynamic stimulation. MSC-derived TEMs exhibited approximately 2–3-fold increases in hydroxyproline content under hydrodynamic conditions compared to static culture (Figure 4). For example, hADMSC matrices increased from ~15 µg/mg (static) to ~30 µg/mg (dynamic), while hUCMSC matrices increased from ~20 to ~40 µg/mg. hBMSC-derived TEMs increased from ~10 to ~20 µg/mg. HDFB-seeded matrices demonstrated the highest collagen levels overall, reaching ~50 µg/mg under hydrodynamic conditions. Statistical analysis confirmed significant effects of culture condition and a significant cell type × culture condition interaction.

Histological nuclei count paralleled DNA findings, with HDFB-derived matrices exhibiting higher cellular density under both conditions (~350–400 nuclei per field) (Figure 4). MSC-derived matrices displayed lower but variable nuclei counts (~180–300 nuclei per field), with hydrodynamic stimulation contributing to more uniform cellular distribution in certain groups. Together, these data suggest that enhanced ECM accumulation under hydrodynamic conditions reflects increased matrix production per cell rather than simply increased cellularity.

Qualitative and quantitative results show that HDFBs consistently demonstrated the highest overall ECM performance under both static and hydrodynamically stimulated conditions, particularly in terms of collagen content and matrix density. Among MSC populations, hUCMSCs and hADMSCs exhibited moderate to high ECM performance, with improvements under hydrodynamic stimulation, whereas hBMSCs showed comparatively lower matrix production across conditions. Collectively, this summary highlights the combined influence of cell source and hydrodynamic stimulation on ECM quantity and structural maturation.

## 4. Discussion

In tissue engineering and regenerative medicine, the concept of an “ideal” extracellular matrix extends beyond maximizing matrix quantity and instead centers on achieving a set of functional characteristics that support long-term tissue integration and regeneration [15]. An effective ECM should provide sufficient mechanical integrity to withstand physiological loading, while remaining permissive to cellular infiltration, vascularization, and matrix turnover [3,37]. Equally important are collagen maturity, fiber organization, and spatial distribution, as these parameters govern not only tissue mechanics but also cell–matrix signaling and remodeling dynamics [38]. In regenerative strategies that rely on in situ remodeling, the implanted matrix serves as a transient instructive template whose ultimate performance is defined by its interaction with the host environment, including immune modulation, degradation kinetics, and replacement by newly synthesized tissue [39]. Thus, ECM quality, characterized by appropriate collagen composition, fiber architecture, and adaptability rather than sheer abundance, emerges as the key determinant for functional regeneration.

This study demonstrated that dynamic mechanical culture markedly enhanced both the quantity and structural quality of ECM produced by MSCs and dermal fibroblasts. Across all cell types, hydrodynamically stimulated TEMs accumulated significantly more collagen and GAGs than their static counterparts, with hydroxyproline content increasing 2–3-fold under hydrodynamic conditions. Notably, this enhancement was not accompanied by an increase in DNA content, indicating that dynamic stimulation promoted greater ECM production per cell rather than cell proliferation. These findings are consistent with previous reports showing that mechanical cues stimulate matrix synthesis and improve TEM properties [40,41,42,43]. In addition, previous studies suggest that mechanical stimulation may influence ECM production through signaling pathways such as TGF-β/SMAD, mechanosensitive ion channels including Piezo1, and cytoskeletal tension–dependent transcriptional regulators such as YAP/TAZ [44,45,46,47]. Beyond quantity, hydrodynamically stimulated culture also improved ECM quality, particularly collagen maturation. Histological and polarized light analyses revealed that dynamically stimulated TEMs developed dense, collagen-rich matrices with fewer scaffold remnants, whereas static cultures remained thin and poorly filled. Under polarized light, dynamic samples exhibited a predominance of thick, strongly birefringent collagen fibers, whereas thin, weakly birefringent fibers dominated static samples. Because Picrosirius Red birefringence reflects collagen fiber thickness, packing, and organization, these results indicate enhanced collagen fiber maturation and organization under hydrodynamic stimulation rather than changes in collagen subtype. This observation is consistent with reports linking mechanical loading to increased collagen crosslinking, fiber stabilization, and hierarchical assembly [48,49].

A central finding of this study is that the origin of MSCs strongly influences their ECM-forming capacity, particularly under hydrodynamically stimulated culture. HDFBs, whose primary physiological role is ECM production, generated the highest collagen and GAG levels. This observation is consistent with their well-established matrix-producing phenotype and with previous studies describing their high expression of structural ECM proteins and responsiveness to profibrotic mediators such as TGF-β [50,51,52,53,54].Taken together, these results emphasize that dynamic mechanical cues are important not only for increasing ECM deposition but also for driving collagen maturation.

Among the MSC populations, we observed the hierarchy hUCMSC ≥ hADMSC > hBMSC in ECM-forming performance. Under hydrodynamically stimulated culture, hUCMSCs and hADMSCs formed thicker, more uniformly distributed collagenous matrices than hBMSCs, consistent with previous tendon and cartilage engineering studies [55,56]. The weaker ECM formation by hBMSCs may relate to their lower proliferative activity and their propensity toward osteochondral or fibrocartilaginous programs, which favor a different ECM composition rather than a dense fibrous collagen I network. In contrast, hUCMSCs and hADMSCs have been reported to exhibit higher proliferative capacity, reduced senescence, and enhanced mechanosensitive responsiveness, and pathways such as integrin–FAK and YAP/TAZ signaling have been implicated in regulating matrix deposition in MSCs [57,58,59]. Together, these in vitro data indicate that cell origin is not a neutral variable but a decisive determinant of ECM quantity and distribution, with HDFBs and hUCMSCs representing the most promising matrix-producing sources within the experimental setting of this study.

Previous studies have shown that MSCs and dermal fibroblasts share substantial transcriptional overlap and even convergent differentiation potential [60,61], and our findings support this to a degree: under dynamic stimulation, hUCMSCs approached fibroblasts in their ability to generate collagen-rich tissue. However, dermal fibroblasts still produced the highest absolute ECM amounts and the most uniformly distributed matrix, consistent with their native role as professional matrix builders in connective tissues. hUCMSCs, derived from neonatal perinatal tissue, possess high proliferative capacity, low senescence, and have been reported to exhibit higher proliferative capacity and enhanced expression of ECM-related and mechanosensitive genes [62,63]. In contrast, hADMSCs and hBMSCs, isolated from adult donors, may display age-associated declines in biosynthetic and mechanosensitive capacity [64,65] and, it could contribute to their reduced ECM-forming performance in this study. On the other hand, ECM generated by dermal fibroblasts, while rich in collagen, has been associated with limitations in regenerative applications. Fibroblast-derived ECM is often dense and compact, with high collagen content but reduced structural plasticity, which can hinder cell infiltration, vascularization, and dynamic remodeling features important for successful functional regeneration and host integration [54,66]. Moreover, in wound healing and pathological contexts, excessive fibroblast ECM deposition is linked to fibrotic matrix formation rather than tissue remodeling [67,68], indicating that high ECM abundance per se is not always advantageous. In contrast, ECM derived from MSCs has been shown to possess greater bioactivity and versatility, supporting cell proliferation, modulating immune responses, and promoting more balanced matrix remodeling. MSC-derived ECM can enhance adhesion and proliferation of multiple cell types [69] and maintain stemness or direct lineage differentiation in vitro [70]. The ability to generate a dense, mature ECM in vitro has major implications for the development of functional grafts for cardiovascular and load-bearing soft tissue repair.

Native tissues such as heart valves, vascular grafts, tendons, and ligaments rely on a hierarchically organized collagen-rich ECM to provide mechanical strength, durability, and appropriate mechanotransduction, making both collagen quantity and collagen maturation critical determinants of performance [15]. In this context, dECM is widely regarded as the benchmark for high-quality biomimetic scaffolds, as it preserves the native biochemical composition, ultrastructural organization, and matrix-bound signaling cues that regulate cell behavior and tissue remodeling [71]. Recent advances in ECM-inspired biomaterials emphasize that dECM supports cell adhesion, mechanosensing, immune modulation, and tissue-specific remodeling more effectively than simplified or immature matrices, highlighting that ECM quality rather than mere abundance governs regenerative outcomes [72,73]. Our findings further support this concept by showing that dynamic mechanical conditioning and the selection of mechano-responsive MSC sources together enhance ECM organization and maturation in TEMs, yielding matrices with structural features approaching those of native tissues [39,74].

While the present study provides comparative insight into ECM deposition and maturation across cell sources and culture conditions, several limitations should be acknowledged. The matrices were evaluated at an early stage of TEM development (21 days), and although clear differences in matrix deposition and organization were observed, ECM remodeling is a dynamic and progressive process that may further evolve over extended culture periods [75]. Analyses were based on defined time points, and real-time monitoring of bidirectional cell–ECM feedback mechanisms was not performed. Hydrodynamic stimulation was implemented via orbital shaking; although biological effects were evident, time-dependent mechanical properties such as scaffold viscoelasticity and stress-relaxation behavior were not directly characterized. As biological matrices exhibit dynamic mechanical responses that can influence cell morphology, migration, and differentiation, future studies integrating rheological measurements and degradation-dependent mechanical assessment would provide additional mechanistic insight. In addition, the study focused on phenotypic ECM outcomes without direct functional validation of specific mechanotransduction pathways (e.g., integrins, DDRs, CD44, syndecans, or YAP/TAZ), and other biochemical regulators known to influence matrix production, such as TGF-β1 and ascorbic acid (vitamin C), were not independently modulated [76,77]. Finally, ultrastructural cell–matrix interactions were evaluated using standard histological approaches, and higher-resolution imaging would allow more detailed characterization of nanoscale adhesion and scaffold–cell interface dynamics.

## 5. Conclusions

In this work, we compared the ECM-producing performance of different human MSC sources and dermal fibroblasts under static and hydrodynamically stimulated culture on PGA/P4HB scaffolds. Hydrodynamic stimulation was associated with increased collagen and GAG deposition and with more advanced collagen fiber organization across all cell groups. As a result, dynamically cultured TEMs displayed a denser, better-organized, and suggested improved ECM organization than their static counterparts. At the same time, the origin of MSCs significantly influenced outcomes. hUCMSCs and hADMSCs were far more effective than hBMSCs in generating both a higher ECM deposition and improved structural organization, with collagen networks that, under dynamic loading, showed matrix organization more comparable to fibroblast-derived TEM. hBMSCs, in contrast, consistently produced the least ECM and showed limited collagen maturation, even under stimulation. These findings underscore that both biophysical culture conditions and intrinsic cell properties jointly determine ECM output, collagen maturation, and ECM deposition and structural organization. These findings may inform the selection of cell sources and culture conditions for TEM, where enhanced ECM deposition and organization are desired. Overall, our study provides evidence-based guidance for the design of next-generation regenerative implants: by combining appropriate cell sources with fluid-induced mechanical cues, faster and more complete matrix maturation can be achieved in vitro. These findings guide optimizing ECM deposition and structural organization in vitro and establish a foundation for future functional and in vivo studies.

## Figures and Tables

**Table 1:** Thickness of TEMs after 21 days under static and dynamic conditions. Values represent mean ± standard deviation (µm) for each cell type.

Cell Type	Static Condition (Mean ± SD, µm)	Hydrodynamic Condition (Mean ± SD, µm)
HDFB	331.233 ± 37.818	353.883 ± 57.089
hUCMSC	330.097 ± 29.817	382.687 ± 39.187
hADMSC	439.027 ± 74.349	597.459 ± 40.665
hBMSC	357.733 ± 41.943	517.205 ± 40.165

## Data Availability

The data presented in this study are available on request from the corresponding author.

## Supplementary Materials

The following supporting information can be downloaded at https://www.mdpi.com/article/10.3390/biomedicines14030560/s1. Table S1: hMSC donor characteristics. Figure S1. Qualitative immunohistochemical analysis of ECM components in TEMs after 21 days under static and hydrodynamically stimulated culture.
