# Supplementary material for: MSC Origin and Biomechanical Conditioning Determine ECM Maturation in Tissue-Engineered Matrix
Source: Biomedicines. 2026 Feb 28;14(3):560. doi: 10.3390/biomedicines14030560 (PMC13023458; doi:10.3390/biomedicines14030560)
Supplement: Supplementary file 1 [file biomedicines-14-00560-s001.zip › biomedicines-4103796-supplementary.pdf]

Supplementary Table S1: *hMSC donor characteristics*

**Cell source**	**Cell type**	**Sample ID**	**Sex**	**Year of birth**	**Passage**
Adipose tissue	hADMSC	A5	Female	1970	4 ± 2
Bone marrow	hBMSC	B7	Female	1950	2 ± 1
Wharton's jelly	hUCMSC	W1	Female (child)	1956 (mother)	5 ± 1

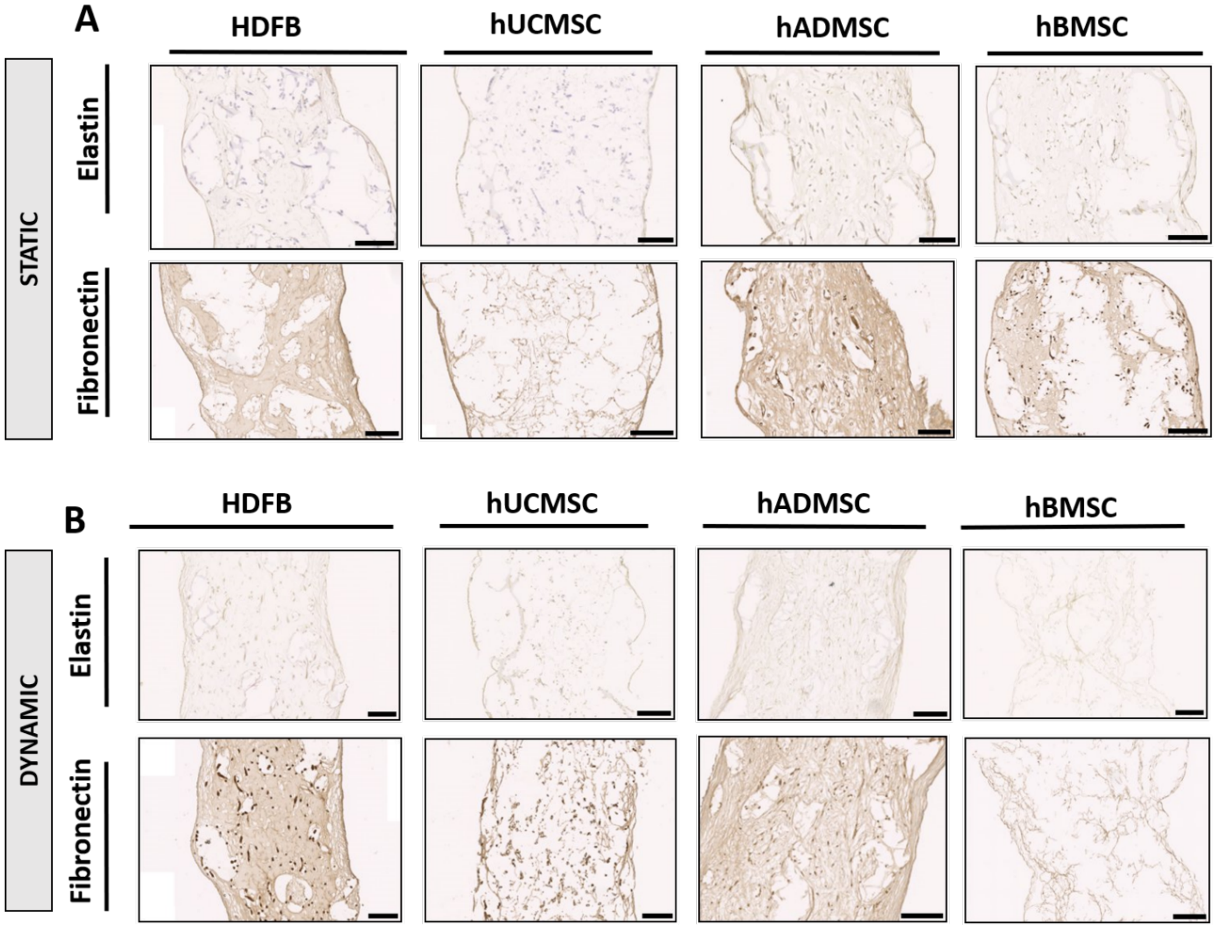

Supplementary Figure S1. Qualitative immunohistochemical analysis of non-collagenous ECM components in TEMs after 21 days under static and hydrodynamically stimulated culture. *Representative sections stained for fibronectin and elastin fibers (Elastica van Gieson, EvG) are shown for each cell source under static and hydrodynamically stimulated conditions. (A) Static cultures exhibited less uniform distribution. (B) Hydrodynamic-stimulated cultures demonstrated more ECM deposition and improved matrix organization. Scale bars: 100 µm.*
